# Thrombopoietin Receptor Agonists: Can These Be the Future Answer to the Deadly Thrombocytopenia in Dengue Fever?

**DOI:** 10.7759/cureus.4361

**Published:** 2019-04-01

**Authors:** Sayak Roy

**Affiliations:** 1 Internal Medicine, Calcutta Medical Research Institute Hospital, Kolkata, IND

**Keywords:** thrombopoietin receptor agonists, thrombocytopenia, dengue, immune thrombocytopenic purpura

## Abstract

Dengue is considered the most prevalent mosquito-borne viral disease worldwide and sometimes turns out to be life-threatening. Thrombocytopenia is frequently observed in mild and severe cases of dengue. Severe thrombocytopenia, with a platelet count much below the normal range and hemorrhagic manifestation, is considered a fatal consequence of dengue that needs proper and timely management. The development of the dengue vaccine is quite challenging due to the existence of four different serotypes of the virus. Currently, neither a specific antiviral therapy nor a vaccine is available, and the common treatment modalities include fluid replacement therapy and platelet transfusions. Besides dengue, thrombocytopenia is correlated with many other diseases, particularly immune thrombocytopenic purpura (ITP). Thrombopoietin receptor (TPO-R) agonist, which is responsible for increasing platelet count, is a novel treatment option for chronic ITP patients. At present, two TPO-R agonists - eltrombopag and romiplostim - approved by the US Food and Drug Administration (USFDA) have been successfully used for the treatment of chronic ITP and other thrombocytopenic conditions. However, to date, only a single case study reported the use of romiplostim to enhance the platelet count in a myeloma patient suffering from dengue-associated thrombocytopenia. The objective of this review is to propose to the medical fraternity to consider using these TPO-R agonists to treat dengue hemorrhagic patients with thrombocytopenia and to conduct relevant researches to find out the usefulness of these molecules. This review is completely based on hypotheses and articles showing the positive response with romiplostim in dengue after going through a web-based search on various search engines. Furthermore, this review highlights the need for good-quality, randomized controlled trials and meta-analyses to detect the safety and efficacy of romiplostim and eltrombopag therapy for patients suffering from dengue-related thrombocytopenia.

## Introduction and background

Dengue is an infectious, arboviral disease of humans transmitted by mosquitoes [1]. According to the World Health Organization (WHO), dengue is a major public health challenge worldwide, with a higher incidence in tropical and subtropical countries [2]. Global dengue occurrence has increased 30-fold between 1960 and 2010 along with the prevalence of severe dengue cases [3]. The global resurgence of dengue over the last five decades mostly resulted from demographic and societal changes, including a boom in population growth rate, unplanned and uncontrolled urbanization, deterioration of waste management systems, global warming, inefficient mosquito control, changes in public health policy, excessive cross-country air travel, and, most importantly, evolution of dengue virus (DENV) with the emergence of higher virulent strains [2,4-5]. More than 2.5 billion people of the world’s population reside in high-risk dengue-transmission areas, with approximately 400 million infections occurring annually and about a 5%-20% mortality rate [2]. Currently, more than 125 countries, including Europe and the United States (US), are known to be dengue-endemic [2,5] with almost 75% of the dengue-exposed population inhabiting the Asia-Pacific region [3]. The anticipated yearly dengue burden of 750,000 disability-adjusted life years (DALYs) is greater than the global burden of 17 other diseases. Dengue has been proclaimed as a priority infection by WHO, United Nations International Children's Emergency Fund (UNICEF), and World Bank. Yet, no antiviral drugs or licensed dengue-specific vaccines exist at present to prevent the infection [6].

Dengue virus

Dengue virus (DENV) is a single-stranded ribonucleic acid (RNA) virus belonging to the family Flaviviridae, which includes the Yellow Fever virus, West Nile virus, and around 70 other viruses [1,4]. There are four antigenically distinct dengue virus serotypes, known as DENV-1, DENV-2, DENV-3, and DENV-4, belonging to the genus Flavivirus [4-5]. Each individual DENV serotype triggers a distinctive host immune response and has been responsible for dengue epidemics [7-10]. Additionally, infection with one DENV serotype leads to the development of lifelong immunity toward that particular strain but no cross-protective immunity towards other serotypes [5]. Hence, in a dengue-endemic area with multiple co-circulating DENV serotypes, there is a probability of sequential infection from two, three, or even four serotypes throughout the life of a person [4].

Clinical manifestations of dengue

Dengue is a serious illness with a wide spectrum of clinical manifestations. There are generally three phases of illness: i) febrile, ii) critical, and iii) defervescence or recovery phase. Normally, following a bite from an infected mosquito, a person suffers from fever [11]. This non-specific febrile state, referred to as dengue fever (DF), is accompanied by general malaise, weakness, severe muscle and joint pain, retro-orbital pain, headache, and often skin rashes [1,12]. Typically, this non-specific DF is relatively benign and self-limited; the virus gets controlled, the fever subsides, and the patients generally recover within a few days [11-12]. Nonetheless, in about 1%-5% of cases, more severe forms of the disease, including hemorrhage, plasma leakage, edema, shock, and hypotension, might occur. This syndrome is called dengue hemorrhagic fever (DHF), which in extreme cases, results in dengue hemorrhagic shock (DHS) or dengue shock syndrome (DSS) [11]. The laboratory tests at this stage indicate higher levels of the liver enzyme, leukopenia, and thrombocytopenia [12]. DHF can be life-threatening, with a 20% fatality rate [11]. Hence, early clinical recognition followed by anticipatory treatment is crucial for the management of patients with DHF/DHS [13]. In fact, studies reported that efficient management primarily with fluid replacement resulted in spontaneous recovery from capillary leakage followed by full recovery and, in turn, reduced the fatality rate below 1% [11,14].

Severity of different serotypes

The four DENV serotypes share about 65% of their genome and differ by 30%-35% in the primary amino acid sequence [7-8]. The DENV genome encodes three structural proteins, such as precursor membrane (prM), envelope (E), and capsid (C), as well as seven non-structural proteins, including NS1, NS2A, NS2B, NS3, NS4A, NS4B, and NS5 [3]. According to the virus virulence hypothesis, certain DENV strains cause more severe diseases than others, mostly due to the differences in the nucleotide sequences of their envelope protein genes, which confer their virulence [9-10]. This might have resulted in some variations in the clinical manifestations of dengue caused by different serotypes [10]. The pathogenicity of dengue, which is quite complicated, depends on several factors, including viral virulence factors, host immune responses [3], and the intra-epidemic evolution of the circulating DENV [9]. The primary DENV-1 infection is considered to be more severe than the primary DENV-2 and DENV-3 infections. In contrast, a secondary DENV-2 infection results in more severe clinical manifestations as compared to other serotypes [10]. In fact, a secondary dengue infection with heterologous serotypes is more critical than primary infection, in most cases, due to the antibody-dependent enhancement (ADE) mechanism [8]. Immunogenic studies of different DENV-serotypes actually determined that NS4A, NS4B, and E-peptides of DENV-2 and DENV-3 viruses trigger more cytokine responses of the host following secondary infection, leading to severe dengue pathogenesis compared to other serotypes. DENV-4 is the least immunogenic among all the serotypes [8].

Transmission cycle of DENV

Dengue virus is maintained in nature and prefers blood-sucking mosquito vectors and human hosts. The transmission of the virus occurs via Aedes mosquitoes, particularly Aedes aegypti (A. aegypti), which is the principal vector and is highly adapted with humans in the urban, domestic environment [4]. Following the bite of a DENV-infected A. aegypti, a person remains in a latent phase for three to 14 days and subsequently develops acute fever and other specific or non-specific signs or symptoms. In this phase, the virus circulates in the peripheral blood of the infected individual and gets transmitted to another uninfected individual by the vector [4].

## Review

Dengue hemorrhagic fever and dengue shock syndrome

**Figure 1:**
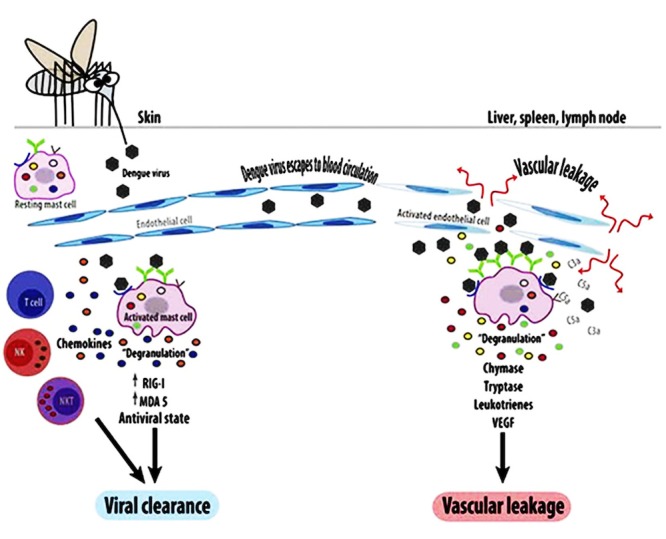
Pathogenesis of dengue virus infection.

The clinical features of DHF can be distinguished from DF on the basis of three pathophysiological phases: i) febrile phase, which is viremia-driven high-grade fever, along with facial erythema, lasting for two to seven days, ii) critical/plasma leak phase with increased vascular permeability, leading to varying degrees of plasma leakage into pleural and abdominal cavities, low pulse pressure, cyanosis, and hepatomegaly, and iii) convalescence or reabsorption phase characterized by sudden cessation of plasma leakage along with the reabsorption of extravasated plasma and fluids [2,13]. Figure 1 depicts the dengue pathogenesis diagrammatically. According to WHO, DHF is classified into four grades depending on severity. Grades I and II are characterized by vasculopathy with the absence (grade I) and presence (grade II) of spontaneous bleeding of the skin and mucous membrane due to the fragility of blood vessels, dysfunction of platelets leading to defective coagulation pathways, thrombocytopenia, and slight capillary leakage [2,12,14-15]. Grades III and IV are characterized by increased vascular permeability, extravasation of fluids, dehydration, no detectable pulse or pressure, and hypovolemic shock, leading to multiorgan failure and, ultimately, death. DHF grades III and IV are together referred to as DHS or DSS, which requires prompt management [14-15]. Sudden onset of shock usually occurs between the third and fifth days of illness and lasts for 24-48 hours [16]. The pathophysiology of DHF/DHS is highly complicated and occurs either due to infection with more virulent DENV strains or more frequently due to a secondary dengue infection [5] that results in abnormal and enhanced host-immune responses [15]. Primary DENV infection produces serotype-specific antibodies without providing any immunity towards unexposed serotypes [8]. Subsequent secondary infection with heterologous DENV-serotype results in the formation of antibodies, which instead of neutralizing the virus, form virus-antibody complexes with an increased affinity towards FcγRI and FcγRII on the macrophage cells. This antibody-dependent enhancement (ADE) process heightens viral entry into cells and viral replication, thereby enhancing the viral load in each cell [8,11]. ADE also generates pro-inflammatory responses by activating the cytotoxic T-cells, which release a variety of cytokines, including TNF-α, Interleukin (IL)-2, 6, 10, 5, 4, and IFN-γ, which removes virus-infected cells. This "cytokine storm" is responsible for endothelial damage and the altered functionality of endothelial cells thus leading to vascular permeability, capillary leakage, and development of shock [15].

Thrombocytopenia in dengue

Thrombocytopenia or low platelet count is commonly observed in patients with mild or severe cases of DENV infections and correlates with clinical outcomes. In this condition, the platelet count drops to even <30,000 platelets/uL, which is much below the normal range of 150,000-450,000 platelets/uL and is usually responsible for bleeding in some patients. Thrombocytopenia generally occurs during three to seven days of febrile state [17]. Primarily, one of two factors: i) decreased platelet production in the bone marrow (BM) or ii) increased immunogenic destruction of platelet along with hemophagocytosis or platelet clearance from peripheral blood causes thrombocytopenia [18]. Platelets are small, anucleated cells derived from megakaryocytes and are responsible for coagulation and homeostasis as well as intracellular communication resulting in inflammatory and immunomodulatory activities [19]. A reduction in platelet count results in excessive bruising (purpura), bleeding, and petechiae [12,18]. Several studies revealed that DHF is marked by the early hypocellularity of BM with a significant drop in the production of all cell lines because DENV directly affects medullary stroma and hematopoietic progenitor cells [12]. In contrast, in the defervescence period, the BM becomes hypercellular, with increased production of the precursor cells of all three medullary cell lines and thus the platelet count returns to normal [12]. Even though reduced platelet production is one of the reasons for thrombocytopenia, enhanced platelet destruction and clearance is mostly involved in rapidly induced thrombocytopenia in dengue patients [20].

**Figure 2:**
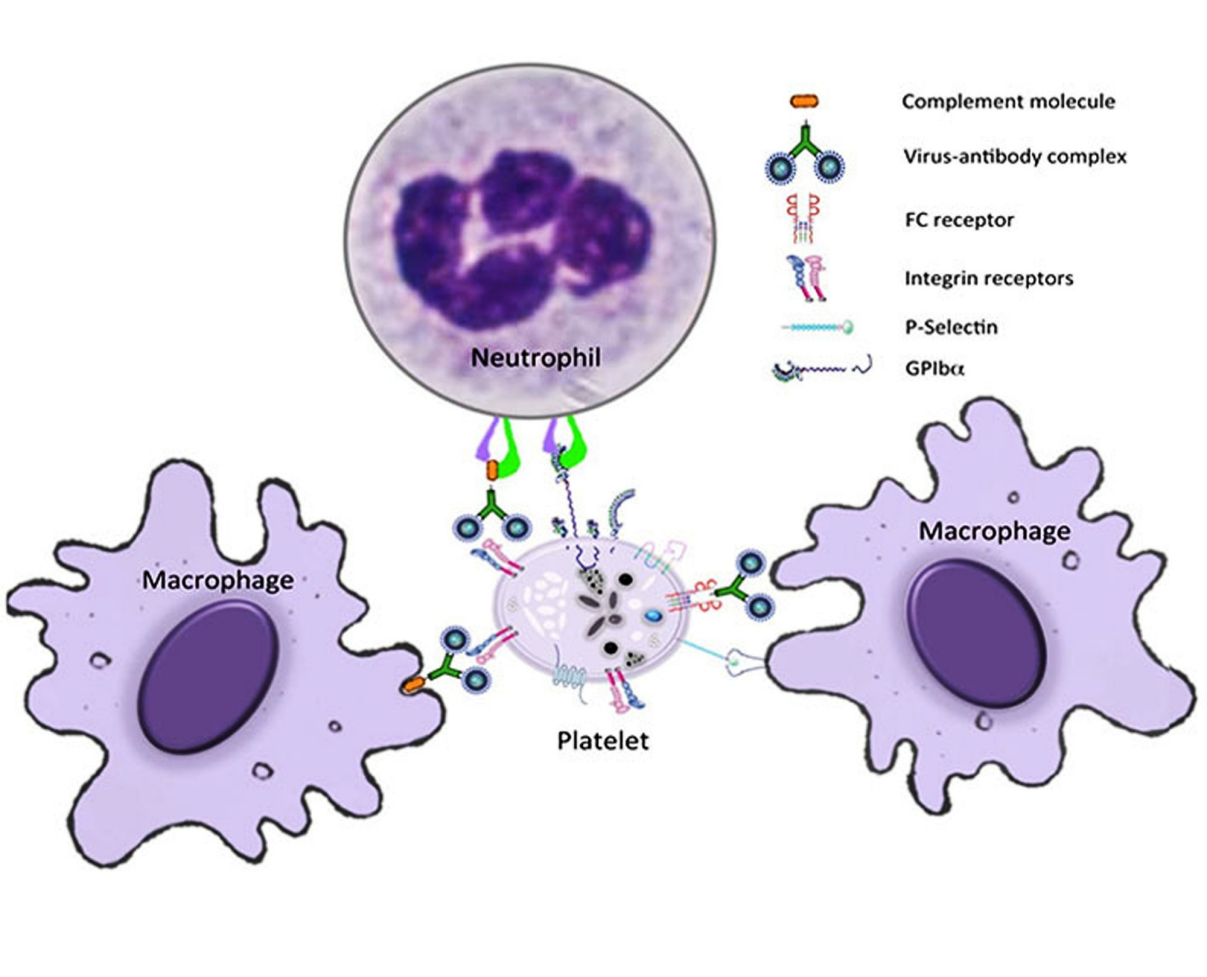
Immunological destruction of platelets. Platelets can interact with macrophages and neutrophils at the infection site and/or in the spleen through immuno-complexes or directly by cellular-ligand interactions. These interactions lead to either platelet sequestration or platelet destruction mediated by the immune system.

DENV infects different cell types, including monocytes, macrophages, skin Langerhans cells, dendritic cells, circulating platelets, and their progenitors (megakaryocytes), in the BM [3]. Platelet destruction and subsequent clearance from the periphery results from the complex interplay of the humoral, cellular, and innate immune responses of the host [2]. The binding of antibody-opsonized DENV with FcγRII on platelets tags the platelets for depletion by the immune cells. Additionally, DENV-NS1-specific antibodies often cross-react with platelet-expressed autoantigens and destroy the platelets. Immunoglobulin M (IgM) class antiplatelet antibodies also abolish platelets [12,18]. Another significant mechanism of platelet destruction in dengue is platelet activation [17]. The mechanism underlying platelet activation is still not known completely. The exposure of platelets to DENV-infected endothelial cells causes platelet activation [21]. Activated platelets are deposited on micro-vessels and phagocytosed by macrophages [17,21]. The platelets in dengue patients with thrombocytopenia usually show signs of activation due to the higher expression of surface phosphatidylserine, activated apoptosis caspase cascade, and mitochondrial dysfunction [21], which, in turn, leads to their depletion. Platelets are also activated by antibodies, cytokines, activated endothelium, coagulation factors, as well as the complement system [21]. The formation of the C3 and C5b-9 complement complex by activated platelets and the consequent binding of the complex to the platelets result in lysis and clearance of platelets [17]. Moreover, activated platelets express increased P-selectin, which mediates the interaction of platelets with different cell types, such as leukocytes, endothelial cells, monocytes, neutrophils, and hematopoietic progenitor cells, leading to the formation of platelet-neutrophil and platelet-leucocyte aggregates. These complexes release enhanced pro-inflammatory cytokines ensuing platelet phagocytosis [19]. Figure 2 shows the destruction of platelets. The higher rate of platelet phagocytosis by macrophages is detected in patients with secondary DENV infection [22]. Platelet activation inhibitor prostacyclin has been reported to block lysis and the clearance of platelets, indicating that the inactivation of platelets actually prevents their destruction [17]. The degree of thrombocytopenia varies in dengue patients; mostly spontaneous recovery of platelets occurs within a few days, either due to increased platelet production by BM or due to the cessation of immune-mediated platelet destruction [23].

Potential biomarkers for DHF/DSS

Platelets have emerged as important biomarkers for different diseases [19]. Platelet activation is considered a potential biomarker for thrombocytopenia [22]. Platelet activation is associated with the enhanced expression of different molecules, such as activated fibrinogen receptor, lysosomal marker CD63, α-granule marker CD62P, P-selectin, CD-40L, PF4, GPIIb/IIIa, and platelet-neutrophil and platelet-leucocyte aggregates. These molecules are referred to as platelet activation makers and are anticipated to contribute to thrombocytopenia and dengue-associated plasma leakage [19,22]. Additionally, activated platelets induce a "cytokine storm," which is a hallmark of DHF/DSS, and the high levels of circulating cytokines and chemokines can serve as a laboratory tool to predict the disease. In fact, increased levels of IL-6, 10, IFN-γ, MIF, and CCL4 are considered potential biomarkers for severe dengue [24]. Moreover, activated platelets in DENV infection induce endothelial cell activation, therefore, the constituents of activated endothelium in plasma might aid in predicting a severe dengue condition [21].

Besides platelet activation markers, there are other molecules, such as dengue-specific IgE, anti NS1, antiplatelet, and anti-EC antibodies, whose levels are enhanced during dengue and could be used as possible predictors of dengue. In addition, host genetic markers, such as the genes LOC286087, MYOM2, CACNA2D3, PSPH, SMAD5, SLC4A4D, and CD244 molecule, with reduced expression in DHF than in DF can be used as predictors of severe dengue. Furthermore, elevated levels of lipopolysaccharide, aspartate aminotransferase, and alanine aminotransferase might serve as biochemical markers for DHF/DSS [24]. Studies showed that an analysis of combination biomarkers is useful in forecasting a severe dengue condition [24].

Thrombopoietin receptor agonists for the treatment of thrombocytopenia

Thrombopoietin (TPO), also referred to as megakaryocyte growth and development factor (MGDF) or c-MpL ligand, is a hormone constitutively synthesized in the liver [25]. TPO binds with the TPO receptors or c-MpL receptors on the surface of megakaryocytes and controls the process of megakaryocytopoiesis or platelet production by triggering intracellular signaling cascades [25-26]. Binding of TPO to circulating platelets is followed by platelet cycling (production and destruction), which, in turn, regulates the level of plasma TPO [25]. Hence, the TPO level is inversely proportional to platelet production and platelet mass. To treat thrombocytopenia, recombinant TPO (rTPO) was used initially to enhance the production of platelets in patients, but its use was discontinued later due to the production of neutralizing anti-TPO antibodies, which cross-react with endogenous TPOs and result in a return of thrombocytopenia [27]. An effective treatment strategy involves the utilization of TPO receptor (TPO-R) agonists, which mimic the mechanism of action of endogenous TPO on its receptors and induce the activation, proliferation, and maturation of megakaryocytes and thereby enhance platelet production [28]. In 2008, the United States Food and Drug Administration (USFDA) approved two TPO-R agonists: eltrombopag and romiplostim for the treatment of idiopathic thrombocytopenic purpura (ITP) and other thrombocytopenic conditions. Recently, the TPO-R agonists are considered second-line treatments for persistent and chronic ITP patients, who are unresponsive to or show recurrence following first-line ITP therapies [29]. The major advantage of these molecules is that they do not cross-react with endogenous TPOs due to the differences in their binding sites [30]. Thus, TPO-R agonists and endogenous TPOs are able to operate concomitantly, producing an additive cell-signaling effect and eventually yielding more platelets [31].

Eltrombopag

Eltrombopag is a small molecular weight, non-peptide, oral TPO-R agonist that binds with the transmembrane domain of a TPO receptor and induces the Janus Kinase/Signal transducer and activator of transcription pathway, thus stimulating megakaryocytopoiesis with a significant increase in platelet production in patients suffering from ITP [30]. A dose-ranging study on eltrombopag by Bussell et al. (2007) displayed that 50 or 75 mg of the drug was effective for the short-term treatment of chronic ITP, with a platelet count reaching ≥50,000/mm^3^ and cessation of bleeding in 80% patients within two weeks, suggesting the hemostatic efficacy of the newly formed platelets. Adverse effects were similar in eltrombopag receiving groups and placebo groups and no dose-limiting toxicity was recorded [32]. A study by Kim et al. (2015), involving eltrombopag-treated ITP patients from 2009 to 2014, revealed an excellent treatment outcome with 72% patients achieving a target platelet count of ≥50,000 cells/mL within a median time of 16 days. The starting dose of 25 mg/day was found to be sufficient for most patients to attain the target platelet level, but doses of 50 mg/day or even 75 mg/day were required in some cases. The platelet count had returned to baseline within two weeks upon eltrombopag termination [30]. Multiple clinical trials, such as RAISE (a study in second-line metastatic colorectal cancer), REPEAT (repeat exposure to eltrombopag in adults with idiopathic thrombocytopenic purpura), EXTEND (eltrombopag extended dosing study), were conducted to study the safety and efficacy of the drug in treating thrombocytopenia [33]. These studies detected mostly mild to moderate side effects of eltrombopag, including headache, dizziness, muscle ache, nausea in most patients, and, very rarely, serious effects, such as vascular occlusions, myocardial infarction, stroke, cataract, and rebound thrombocytopenia (upon eltrombopag withdrawal), were noticed [33-34]. An elevation of alanine aminotransferase (ALT) has been reported as one of the most common adverse effects of eltrombopag in patients with ITP. However, this hepatotoxic effect was transient and mild, with the returning of the ALT level to the normal range upon discontinuation of eltrombopag and without any recurrence following drug re-administration [30]. The long-term use of eltrombopag has not been associated with severe bleeding or significant change in BM [30] or any carcinogenic effect [33]. Eltrombopag is predominantly eliminated through feces with only 31% of the metabolized drug excreted via urine, thus requiring no dose adjustments for patients with renal impairment [33]. Both the EXTEND and REPEAT trials identified that the relapse of ITP on prolonged discontinuation of eltrombopag can be reversed by restarting the drug at the previous dose. Eltrombopag therapy is not recommended for patients of age 18 years or younger due to an insufficiency of clinical data [33]. In general, eltrombopag is a quite well-tolerated drug with a decent safety and efficacy profile and can be used for the treatment of splenectomized ITP patients (platelet count <40,000/mm3), who are unresponsive to corticosteroids and immunoglobulins [33].

Romiplostim

Romiplostim is another USFDA-approved TPO-R agonist used to treat thrombocytopenia. It is a recombinant Fc-peptide fusion protein with two domains: a peptide domain consisting of 14 amino acid peptides and a carrier antibody with a crystallized fragment domain. The Fc fragment of romiplostim competes with endogenous TPO to bind to the extracellular domain of the TPO receptor and activates the intracellular thrombopoiesis pathway. Romiplostim has no sequence homology with endogenous TPO. The endothelial recirculation of the crystallized domain elongates the half-life of the polypeptide [34]. Subcutaneous administration of romiplostim increases platelet count and reduces bleeding episodes in ITP patients [35]. With an aim to evaluate the efficacy of romiplostim by measuring the stable platelet response and safety of treatment, two parallel placebo-controlled Phase III trials, involving splenectomized and non-splenectomized ITP patients, with mean three platelet count ≤30 X 109/L were conducted. The patients were subjected to 2:1 subcutaneous romiplostim injections or placebo every week for a period of 24 weeks [36]. Results revealed that about 38% splenectomized patients and 60% nonsplenectomized patients had a durable platelet response in contrast to none in the placebo-treated group (Table 1), with an overall platelet response rate of 88% in splenectomized and 79% in nonsplenectomized patients. The platelet count of 50 X 109/L was achieved in 14-15 weeks. Eighty-seven percent of romiplostim-treated patients had discontinued concurrent ITP therapy as compared to 38% in the placebo-received group. No antibodies against romiplostim or thrombopoietin were produced. Side effects were alike in both the romiplostim and placebo-treated groups [35-36]. Only two romiplostim-treated patients reported adverse reactions involving increased bone marrow reticulin in one patient and peripheral disease and atrial fibrillation in another patient. However, bone marrow reticulin returned to the baseline level on the discontinuation of romiplostim [35]. In another open-label extension study from August 2004 to January 2010 involving adult ITP patients receiving romiplostim for about 78 weeks with an average weekly dose of 4 mcg/kg, 94.5% patients were found to maintain a platelet count of ≥50×109/L and about 82% patients could successfully administer romiplostim at home [37]. Mild adverse effects, including headache, fatigue, and nasopharyngitis, were reported in 98% of patients; only two patients developed neutralizing antibodies to romiplostim. The frequency of adverse events, such as moderate to severe thromboembolic events, did not rise with time [37].

**Table 1:** Platelet response in placebo-controlled studies.

Outcomes	Study 1*		Study 2*	
	(non-splenectomized patients)		(splenectomized patients)	
	Romiplostim (n=41)	Placebo (n=21)	Romiplostim (n=42)	Placebo (n=21)
Overall platelet response, n (%)	36 (88)	3 (14)	33 (79)	0 (0)
Durable platelet response, n (%)	26 (61)	1 (5)	16 (38)	0 (0)
Requiring rescue therapy, n (%)	8 (20)	13 (62)	11 (26)	12 (57)
Average number of weeks with platelet counts $50×109/L	15	1	12	0
*Kuter et al. (2008) [36]				

Cines et al. (2015) recently provided an integrated analysis on the long-term safety of romiplostim from 14 studies, conducted between July 2002 and June 2011, where patients were on romiplostim treatment for 76 weeks with a dose of 4.2 mcg/kg. The most common side effects of romiplostim were found to be a headache, epistaxis, and nasopharyngitis. The rate of thrombotic events was low. Few patients showed increased bone marrow reticulin; only three patients developed neutralizing romiplostim antibodies but no endogenous TPO antibody [38]. The most serious adverse effects of romiplostim deduced from different clinical studies include rebound thrombocytopenia, increased bone marrow reticulin, and the enhanced proliferation of immunoblot [39]. The patients on continued romiplostim treatment were found to achieve durable platelet response and some patients were even able to reduce the dose over time [35]. Data from multiple studies specify that romiplostim is a well-tolerated, safe, and favorable targeted agent to treat thrombocytopenia with very few significant side effects.

Treatment of thrombocytopenia in dengue patients

Thrombocytopenia, associated with an increased risk of bleeding and life-threatening hemorrhage, is considered as a potential indicator of clinical severity in dengue patients. It reduces the quality of life of the patients. The degree of thrombocytopenia varies in dengue infections, with platelet counts in some patients becoming dangerously low: <10,000 but bleeding yet to occur or platelets <50,000 with bleeding manifestations. While platelet transfusion is used as supportive care for dengue patients with acute thrombocytopenia, the process has several disadvantages, such as it neither stops bleeding nor shortens the time for the cessation of bleeding and is also associated with several adverse reactions (infections and allergy) and even death [22-23]. Hence, platelet transfusion is not considered routine treatment management for thrombocytopenia in dengue patients.

The use of corticosteroids, immunoglobulin, and splenectomy remain mainstays of treatment for thrombocytopenia associated with ITP [40]. However, the WHO did not recommend corticosteroids to treat dengue patients due to their adverse effects [41]. Shashidhara et al. (2013) had detected that a high dose of dexamethasone was unable to raise the platelet count in acute DHF patients [42]. Conversely, anti-D immunoglobulin (effective in producing an Fcγ receptor blockade), which enhances platelet count considerably has been found to be an effective and safe therapeutic option for severe and prolonged thrombocytopenia in dengue patients [43]. Additionally, the intravenous administration of γ-immunoglobulin in DHF patients with acute thrombocytopenia successfully enhanced the platelet to the target range and reduced bleeding episode [44]. An alternative therapy without the toxic effects of drugs has been recommended by a few researchers. This included the use of the Carica papaya leaf extract, which is linked with greater megakaryocyte production and, in turn, with increased platelet production [40]. However, more researches are required to prove the efficiency of all the above-mentioned therapeutic options for dengue-related thrombocytopenia.

Currently, two novel TPO-R agonists - eltrombopag and romiplostim - are successfully used for treating thrombocytopenia with mild to moderate side effects. Despite the increasing popularity of TPO-R mimetics as safe and efficacious molecules to treat ITP and other thrombocytopenic conditions, there seems to be a lack of usage of these molecules in treating deadly thrombocytopenia in dengue patients. Till date, only one study reported the fruitful use of romiplostim to treat dengue-related thrombocytopenia in a multiple myeloma patient with BM hypoplasia, who was unresponsive to the thrombopoietin growth factor oprelvekin as well as platelet transfusion. A subcutaneous administration of romiplostim (4 mcg/kg/week) had raised the platelet count rapidly and the patient was able to maintain > 100X109/L platelets even after 230 days of romiplostim treatment [27]. This interesting finding definitely opens a new therapeutic option for treating thrombocytopenia in dengue. Additionally, the RAISE trial had specified that romiplostim has higher efficiency in treating thrombocytopenia compared to eltrombopag [45-46]. At present, no specific vaccines are available to treat dengue and the mortality rate of DHF/DSS patients is increasing due to the lack of precise treatment. In this context, it is extremely important to focus on therapy that would prove effective in the management of thrombocytopenia related to dengue death.

Future direction

Researches should be directed towards detecting the role of both eltrombopag and romiplostim in improving the platelet counts in severe dengue patients with thrombocytopenia. Good-quality randomized controlled trials, involving large study groups, as well as long-term exposure studies, should be conducted to evaluate the effectiveness of these TPO-R agonists in dengue patients. The safety and efficacy profiles of these drugs in dengue patients should be ascertained in order to decide their effective dosage schedule and detect severe adverse side effects on human subjects, including hepatotoxicity, effects on bone marrow, relapse of thrombocytopenia on drug withdrawal, renal tubular toxicity, potential for causing hematologic malignancies, as well as cataracts [45]. However, the chances of getting these side effects seemed low, as therapies in dengue will be for a short period and adverse effects were mainly seen in long-term care. Furthermore, meta-analysis and systemic reviews should be performed based on the data of different clinical trials to obtain more reliable and comprehensive records on the therapeutic efficacies of TPO-R agonists and their cost-effectiveness for the management of thrombocytopenia in DHF patients. We also need to tease out those groups of DHF patients who might benefit from the new molecules by conducting large studies.

## Conclusions

Dengue has evolved as a global, life-threatening public health concern. Thrombocytopenia is a common clinical manifestation of DENV infection. The recommended mainstay of treatment is mostly supportive, including platelet transfusion and appropriate fluid replacement. Worldwide endeavors have been made to develop new management strategies to fight this fatal complication in dengue patients. The principle treatment objective for severe thrombocytopenia patients is to stabilize the platelet count in order to prevent any bleeding. At present, only a single study reported the reversal of thrombocytopenia using romiplostim in a dengue patient. This remarkable finding, along with the previous evidence on the effectiveness of TPO-R agonists in ITP patients, indicates that it is quite imperative to consider these molecules as a treatment modality for the amelioration of dengue-related deadly thrombocytopenia. To accomplish this, high-quality comparative clinical data are required to analyze the relative efficacy, safety, adverse side effects, and cost-effectiveness of romiplostim and eltrombopag in dengue treatment, as well as their impact on the quality of life of the patients. A deep insight into the importance of these molecules in the treatment of DHF patients with thrombocytopenia is vital. Most importantly, there is nothing to lose in trying these TPO-R agonists in thrombocytopenia management, as this simple step, if it really works, might save millions of critical dengue patients.
